# Excelsior of Agriculture

**Published:** 1876-12

**Authors:** 


### EXCELSIOR OF AGRICULTURE.

A LARGER proportion of farmers fill our madhouses than any other one class of persons in the land. We have before stated the reason of this to be, the monotony of their employment, and want of mental stimulus. But this state of things is rapidly changing, by the influence of *Agricultural Journals*, which are establishing themselves in every section of the country; their tendencies are of a healthful character in many ways. By telling the reason of things, they open up a new world of thought to the cultivator of the soil, which is pursued under the influence of a stimulus the most potent in all lands, that of profit. Just give the most ordinary farmer an inkling of how he may make one acre produce as much as an acre and a half did before, and he will dive into the subject with an avidity quite surprising. Then there is the *pleasure of intelligent cultivation*, which is not inferior in its effect on the whole man, to the satisfaction of increased profits, while it is far purer and more elevating.

Furthermore, the character of these journals, as a class, and I know of not a single exception, is solid, substantial, on the side of virtue, integrity and industry.

